# Can the Pirani Score Predict the Number of Casts and the Need for Tenotomy in the Management of Clubfoot by the Ponseti Method?

**DOI:** 10.5704/MOJ.1803.005

**Published:** 2018-03

**Authors:** A Sharma, S Shukla, B Kiran, S Michail, M Agashe

**Affiliations:** Department of Orthopaedics, Central Railway Hospital, Mumbai, India; ^*^Department of Orthopaedics, KJ Somaiya Medical College and Research Centre, Mumbai, India; ^**^Department of Orthopaedics, Topiwala National Medical College, Mumbai, India; ^***^Department of Orthopaedics, General Hospital of Attica KAT, Kifisia, Greece

**Keywords:** idiopathic clubfoot, Ponseti method, Pirani score, CTEV

## Abstract

**Introduction:** We assessed the role of the Pirani score in determining the number of casts and its ability to suggest requirement for tenotomy in the management of clubfoot by the Ponseti method.

**Materials and Methods:** Prospective analysis of 66 (110 feet) cases of idiopathic clubfoot up to one year of age was done. Exclusion criteria included children more than one year of age at the start of treatment, non-idiopathic cases and previously treated or operated cases.

**Results:** The initial Pirani score was (5.5±0.7) for the tenotomy group and the initial Pirani score was (3.3±1.6) for the non-tenotomy group. There was a significant difference between the initial Pirani score for the tenotomy and the non-tenotomy group with t= -7.9, df= 64 p<0.0001. The tenotomy group had a significantly higher number of casts (four to seven) compared to non-tenotomy group (two to five) t=-10.4, df=64, p<0.0001. Spearman’s rank correlation coefficient was significant and confirmed positive correlation between the initial Pirani score and the number of casts required to correct the deformity (r = 0.931, p<0.0001).

**Conclusion:** Initial high Pirani score suggests the need for greater number of casts to achieve correction and probable need for tenotomy. The number of casts required in achieving complete correction increases with increase in the initial Pirani score. The initial high hindfoot score (2.5-3) signifies the probable need of a minor surgical intervention of percutaneous tendoachilles tenotomy. Based on the initial Pirani score, parents can be informed about the probable duration of treatment and the need for tenotomy.

## Introduction

Congenital talipes equinovarus (CTEV) or clubfoot is a common paediatric condition with a reported incidence of 1-2 per 1000 new born^1^. The Ponseti method is the most popular worldwide for non-surgical correction of idiopathic clubfoot, with excellent long-term outcome (30 years)^2^. Pirani *et al* devised a simple scoring system and reported good intra-observer reliability for their scoring system based on six clinical signs - three each for midfoot and hind foot. Each foot received a midfoot and hind foot score between 0 and 3 and a total score between 0 and 6. Each was scored according to the degree of abnormality (0; being no abnormality, 0.5; moderate abnormality, 1; severe abnormality)^3,4^.

Flynn *et al* further confirmed that Pirani scoring system had very good intra-observer reliability after the initial learning phase. The other popular classification for clubfoot worldwide is the Dimeglio classification^5^. The Ponseti technique remains the most accepted and reliable method of management of clubfoot today and it has also reduced the need for major foot surgery significantly^6^. In a Cochrane review, to compare various techniques, the authors concluded that the Ponseti technique produced significantly better short-term foot alignment compared to the Kite and other traditional techniques^7^. Parents of children being treated for clubfoot are likely to enquire about the duration of treatment and the need for tenotomy. Previous studies have shown variable results in the Pirani scores’ ability to predict the number of casts required and the need for tenotomy^8-12^.

In our study, we have assessed the role of the Pirani score in predicting the number of casts required and the need for tenotomy in the management of clubfoot by the Ponseti method.

## Materials and Methods

The present study was done from July 2012 to March 2015, with approval from the Institutional Ethics Committee for research on human subjects. Prospective analysis of 66 cases of idiopathic clubfoot up to one year of age was done. Exclusion criteria included children more than one year of age at the start of treatment, non-idiopathic cases and previously treated or operated cases. All the cases of CTEV presenting between July 2012 to March 2015 in our hospital and meeting the inclusion criteria were included in the study.

The data was prospectively collected for 66 children (110 feet) after conducting a complete general, systemic and local examination. The severity of the deformity was assessed using Pirani scoring system and the recording of Pirani score was done during each visit by the first author. The third author on a weekly basis did serial casting according to Ponseti technique. The second and third authors together decided the need of tenotomy. All the other authors were blinded to the initial Pirani score except the first author. After achieving 70 degrees of abduction and correction of heel varus, if ankle dorsi-flexion remained <10 degrees, percutaneous tenotomy was performed under local anaesthesia. Cast was applied following tenotomy in full abduction and dorsiflexion for three weeks. After achieving complete correction, a custom-made Steenbeck brace with 70 degrees external rotation on the affected foot and 40 degrees external rotation on the normal foot and 15 degrees bend of the connecting bar to maintain dorsiflexion was given. The size of the splint was determined prior to tenotomy so that the brace could be applied immediately after cast removal. The brace was advised to be worn full time for the first three months and then to use the brace for 12 hours at night and two to four hours in the day for a total of 14 to 16 hours during the 24-hour period until the child was 3 to 4 years of age. During each visit, the corrected foot was examined in detail and strict bracing protocol was advised^7^. Follow-up of minimum one year was available for all cases.

The relationship between the number of casts and the respective initial Pirani scores was assessed using the Spearman’s rank correlation and co-efficient with the level of significance set at p=0.05. The predictor variables to predict number of casts required were the Pirani score and age. The relative importance of the predictor variables to the number of casts was estimated using step-wise multiple regression analysis. Variables with p value less than 0.1 on f-test were not retained in the model.

## Results

**Fig. 1:**
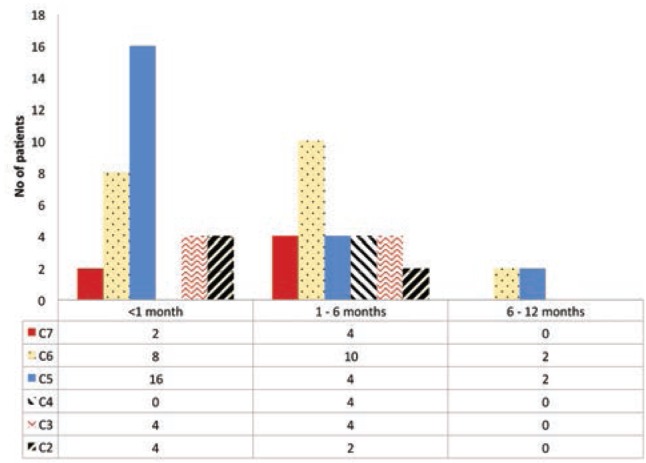
Age versus number of casts required. C represents number of casts.

In our study, the total number of cases treated was 66. The total number of feet treated was 110. Out of the 66 cases, 44 cases had bilateral involvement. In unilateral cases, the right side (14 cases, 64%) was more commonly involved. The minimum follow-up duration was 12 months and maximum follow-up duration was 30 months for cases registered early in the study. Most of the cases (34 cases, 51.5%) were below one month of age. The youngest child in the study was of five days of age and the oldest was of seven months. Males were 52 cases (78.8%) and 14 cases (21.2%) were females; showing a male preponderance. Most of the cases were in the age group of below one month and patients presenting early after birth required lesser number of casts compared to those who presented late (Fig. 1).

Tenotomy was needed in 40 cases (84 feet, 76.3%) and the mean initial Pirani score was 5.02 for 110 feet, with minimum score of 2.0 and maximum score of 6.0. The initial Pirani score was (5.5 ± 0.7) for the tenotomy group and the initial Pirani score was (3.3 ± 1.6) for the non-tenotomy group. There was a significant difference between the initial Pirani score for the tenotomy and the non-tenotomy group with t = -7.9, df=64 p<0.0001.

**Fig. 2:**
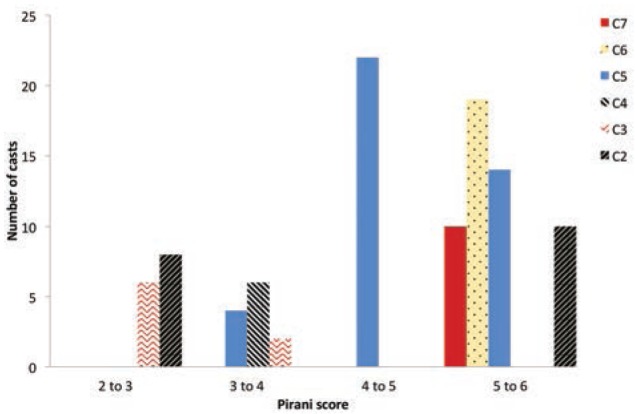
Pirani score versus number of casts required, C represents number of casts.

The mean initial hind foot score was 2.70 for 110 feet, with minimum score of 1.5 and maximum score of 3.0. The mean initial hind foot score was 2.87 for the tenotomy group and the mean initial hind foot score was 2.10 for the non-tenotomy group. There was a significant difference between the mean initial hind foot score for the tenotomy and the non-tenotomy group t = -4.680 and p = 0.00. All 100% of the tenotomy group had an initial hind foot score of >2.5 compared with (57.6%) of the non-tenotomy group (Fig. 2).

The mean number of casts required for 110 feet was 5.10. The number of casts required among the tenotomy group was (5.7 ± 0.8) and among non-tenotomy group was (3.3 ± 1.1). The tenotomy group had a significantly higher number of casts (4 to 7) compared to non tenotomy group (2 to 5) t = -10.4, df = 64, p<0.0001. The number of casts required to achieve complete correction increased with increase in the initial Pirani score (Fig. 2). The majority of the feet in the study had an initial Pirani score between 5 and 6.

Spearman’s rank correlation coefficient was significant and confirmed a positive correlation between the initial Pirani score and the number of casts required to correct the deformity (r = 0.931, p <0.0001). The tenotomy group showed a strong positive correlation between the initial Pirani score and the number of casts required to correct the deformity (r = 0.931, p <0.0001). The non-tenotomy group also showed a strong positive correlation between the initial Pirani score and the number of casts required to correct the deformity (r = 0.95, p < 0.0001).

**Fig. 3:**
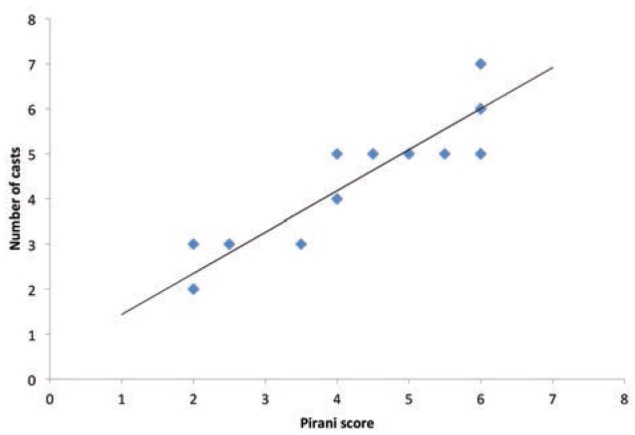
Association between Pirani score and number of casts.

In step-wise multiple regression analysis only the variable ‘Pirani score’ was retained in the model while age was dropped as it was not a significant predictor (P 0.343). The association was positively correlated (Fig. 3) and estimated number of casts = 0.53 + 0.91 (Pirani score). The amount of variance explained in the model r2 was 0.87. The co-efficients are detailed in Table I. The other observations made in our study were: the initial Pirani scores were the same in both feet among all bilateral cases. The deformity was found to be severe (high Pirani score) among bilateral cases (Table II).

**Table I:** Regression model analysis showing coefficients in the regression line with their standard error and significance. Age was dropped out of the model

	**Co-efficient (β)**	**Standard error (β)**	**p-Value**
Constant	0.53	0.22	0.022
Pirani score	0.91	0.05	<0.0001

**Table II:** Summary of patient demographics and treatment outcome between the two groups

	Tenotomy (n=84)	No tenotomy (n=26)
Age (days)	48.6±8.7	51.1±10.8
Gender	42 males, 4 females	10 males, 10 females
Pirani score	5.5±0.7	3.3±1.6
Number of casts	5.7±08	3.3±0.1.1

## Discussion

Congenital idiopathic clubfoot is a common congenital foot deformity, treated by widely accepted and acclaimed Ponseti technique to achieve early correction^13^. Pirani score is the most popular method to track progress to predict the need for tenotomy, and the number of casts of required^8,14^. In the present study, the mean age at initial presentation was 6.7 weeks comparable with Laaveg *et al*^15^ study. Eighty-four feet (76.3%) underwent tenotomy comparable to studies done by Colburn *et al*^16^ and Morcuende *et al*^17^. In our study, the number of casts required to achieve complete correction increased with increase in the initial Pirani score. The association was positively correlated (Fig. 1) and estimated as number of casts = 0.53 + 0.91 (Pirani score). Raju *et al*^18^ showed in their series faster rates of decrease in Pirani score treated by Ponseti technique, and less number of casts with less Pirani score. Dyer PJ and Davis N^8^ in their series showed at least four casts were required for full correction of initial Pirani score of 4. Bor *et al* in their series had mean total Pirani score of 4.7 (2 to 6) and mean number of cast required was six, similar to our study^19^.

In our study, both the tenotomy and non-tenotomy group showed a strong positive correlation between the initial Pirani score and the number of casts required to correct the deformity, confirmed by Spearman’s rank correlation coefficient. Further, the mean initial Pirani score for 110 feet was 5.02. There was a significant difference between the initial Pirani score for the tenotomy group (5.5 ± 0.7) and the non-tenotomy group (3.3 ± 1.6). The mean initial hind foot score was 2.70. There was a significant difference between the mean hind foot score for the tenotomy group (score-2.87) and the non-tenotomy group (score-2.10).

The mean number of casts required to correct the deformity was 5.10 (range 2-7). In the Morcuende *et al* series, the number of casts ranged from one to seven, 90% of the patients required less than five casts for correction^17^. Similarly, Scher *et al*^20^, reported mean number of casts as 5.7 (range 4-9), while Dobbs *et al*^13^, required 4.16 (range 3-7) casts for correction. There was a significant difference between the number of casts required to correct the deformity for the tenotomy group (84 feet; casts- 5.7 ± 0.8) and the non-tenotomy group (36 feet; casts -3.3 ± 1.1). The present study does have its limitations like the short follow-up period, so we have focused only on the importance of Pirani’s scores during the treatment in predicting the number of casts and the need of tenotomy. Only children below age of one year and idiopathic cases have been included in our study to remove confounding factors in syndromic cases.

## Conclusion

Based on this study to assess the importance of Pirani scoring and its role in predicting the number of casts and the need of tenotomy in the management of idiopathic clubfoot by the Ponseti method, in children less than one year, the initial high Pirani score signifies a longer duration of treatment, the need for greater number of casts to achieve correction and probable need for tenotomy. The number of casts required in achieving complete correction increases with increase in the initial Pirani score. The initial high hindfoot score (2.5-3) signifies the probable need for a minor surgical intervention of percutaneous tendoachilles tenotomy. Based on the initial Pirani score, parents can be reliably informed about the probable duration of treatment and the need of tenotomy.

## Conflict of Interest

None of the authors have any potential conflict of interest and no funding or grant was received for the above project.
